# Elevation of MAP17 enhances the malignant behavior of cells via the Akt/mTOR pathway in hepatocellular carcinoma

**DOI:** 10.18632/oncotarget.21506

**Published:** 2017-10-04

**Authors:** Xinhuang Chen, Yan Liao, Yaqun Yu, Pengpeng Zhu, Jun Li, Liling Qin, Weijia Liao, Zhaoquan Huang

**Affiliations:** ^1^ Laboratory of Hepatobiliary and Pancreatic Surgery, Affiliated Hospital of Guilin Medical University, Guilin, Guangxi, P.R. China; ^2^ Disease Prevention and Control Center of Guilin, Guilin, Guangxi, P.R. China; ^3^ Department of Hepatobiliary and Pancreatic Surgery, Affiliated Hospital of Guilin Medical University, Guilin, Guangxi, P.R. China; ^4^ Guangxi Key Laboratory of Molecular Medicine in Liver Injury and Repair, Guilin Medical University, Guilin, Guangxi, P.R. China; ^5^ Department of Pathology, Guilin Medical University, Guilin, Guangxi, P.R. China

**Keywords:** hepatocellular carcinoma, MAP17, progression, Akt/mTOR, prognosis

## Abstract

MAP17, a small non-glycosylated membrane protein, was significantly up-regulated in hepatocellular carcinoma (HCC) tissues in our previous genome-wide microarray analysis. In this study, quantitative real-time RT-PCR and immunohistochemistry were applied to examine MAP17 mRNA and protein expression in primary HCC and matched peritumoral tissues. The disease-free survival (DFS) and overall survival (OS) was estimated using the Kaplan-Meier analysis. The expression of MAP17 was significantly higher in HCC tissues compared to the paired peritumoral tissues at both mRNA and protein levels. High MAP17 expression was positively correlated with gender, distant metastasis, early recurrence (≤ 2 year), and serum alpha-fetoprotein (all *p* < 0.05). Kaplan-Meier analysis showed that the DFS (*p* = 0.004) and OS (*p* = 0.013) in HCC patients with elevated expression of MAP17 were much worse than that in the low expression subgroup. High level of MAP17 was also significantly associated with a high probability of HCC early recurrence after surgical resection (*p* = 0.005). Cox regression analysis indicated MAP17 was an independent prognostic factor for DFS (HR, 1.710; 95% CI, 1.156-2.449, *p* = 0.012) and OS (HR, 1.743; 95% CI, 1.152-2.639, *p* = 0.009) in HCC. Silencing MAP17 significantly inhibited the proliferation, invasion and migration of HCC cells *in vitro*, and decreased the expression levels of Akt, p-Akt (Ser473), mTOR, p-mTOR (Ser2448) and MMP-9. Suggesting MAP17 was a novel diagnostic and prognostic biomarker for HCC patients and promoted HCC cell proliferation, invasion and migration via the Akt/mTOR pathway.

## INTRODUCTION

Hepatocellular carcinoma (HCC) is a severe malignant tumor, ranking as the fifth most common cancer and the third leading cause of cancer-related mortality worldwide [1, 2]. Despite the significant progress made in both diagnostic methods and multimodality treatment for HCC patients, the 5-year survival still improves slowly, which is caused by the high incidences of intrahepatic and extrahepatic metastases and postsurgical recurrence [3–5]. Hepatocarcinogenesis is a multistep process which involves a consequence of genetic and epigenetic changes, and it may activate the oncogenes or inactivate the tumor suppressor genes, leading to the abnormal physiological signals and heterogeneous molecular profiles [6, 7]. Therefore, it is critical to investigate and identify some novel genes involved in HCC initiation and progression, and use them for early diagnosis and prognosis prediction as well as effective therapeutic strategies in HCC.

MAP17 (also known as PDZK1IP1, DD96, SPAP), a small non-glycosylated membrane-associated protein, locates on plasma membrane and Golgi apparatus [8, 9]. Human MAP17 maps to chromosome 1p33, a locus commonly found to be involved in the occurrence and development of human cancer [10]. Growing studies has confirmed that MAP17 is overexpressed in a wide variety of tumors types including prostate, ovarian, cervical, breasts and laryngeal cancer [11–13]. Up-regulation of MAP17 expression increases proliferative capabilities, decreases apoptotic sensitivity and improves migration ability of the tumor cells via increasing the level of reactive oxygen species (ROS) [12, 14]. Interestingly, in our previous genome-wide microarray, we found that MAP17 was significantly up-regulated in HCC tissues analysis. Therefore, it is worth to further explore the relationship between MAP17 and HCC, so as to provide new ideas for early diagnosis, prognostic prediction, and therapeutic target.

In this study, our findings conformed that the MAP17 is upregulated in HCC by quantitative real-time RT-PCR (qRT-PCR) and immunohistochemistry (IHC). The relevance between MAP17 expression and the clinicopathologic features of HCC patients was further explored. Moreover, MAP17 showed a powerful ability to predict the prognosis outcome and early recurrent in HCC patients underwent surgical radical resection.

## RESULTS

### Clinical characteristics of HCC patients

A total of 221 patients underwent surgical radical resection in this study. Table 1 described and summarized clinical characteristics of the patients enrolled in this study. The HCC patients consisted of 192 males and 29 females, with a median age of 50.56±0.80 years. Most of the HCC patients were hepatitis B carriers (81.90%) and had underlying hepatic cirrhosis (90.95%). The mean OS and DFS time was 46.58 months (95%CI, 41.90-51.96) and 40.62 months (95%CI, 35.87-45.82) respectively. The average follow-up time was 46.2 months (median, 22.0 months; range, 1.0 to 84.0 months). The 1-, 3-, and 5-year OS rates were 76.5%, 49.7%, 41.8% respectively.

**Table 1:** Clinical and biochemical data of examined patients

Parameter	Mean±SD
Age (years)	50.56±0.80
Gender: female/male (n)	29/192
Alcohol abuse: yes/no (n)	111/110
Cirrhosis: yes/no (n)	201/20
HBsAg: positive/negative (n)	181/40
Tumor size (range, cm)	8.44±0.34
AFP (ng/ml)	3807.10±1223.02
WBC (109/L)	6.11±0.14
Platelets (109/L)	181.20±5.36
Albumin (g/L)	39.81±0.32
Globuline (g/L)	30.06±0.37
Total bilirubin (μmol/L)	19.06±2.01
Direct bilirubin (μmol/L)	7.80±1.45
ALT (U/L)	46.26±2.99
AST (U/L)	55.68±4.55
ALP (U/L)	99.03±3.79
γ-GT (U/L)	112.50±7.91

HBsAg, Hepatitis B surface antigen; AFP, Alpha-fetoprotein; WBC, White blood cell; ALT, Alanine aminotransferase; AST, Aspartate aminotransferase; ALP, alkaline phosphatase; γ-GT, γ-glutamyl transpeptidase.

### MAP17 expression was up-regulated in HCC tissues

**Figure 1:**
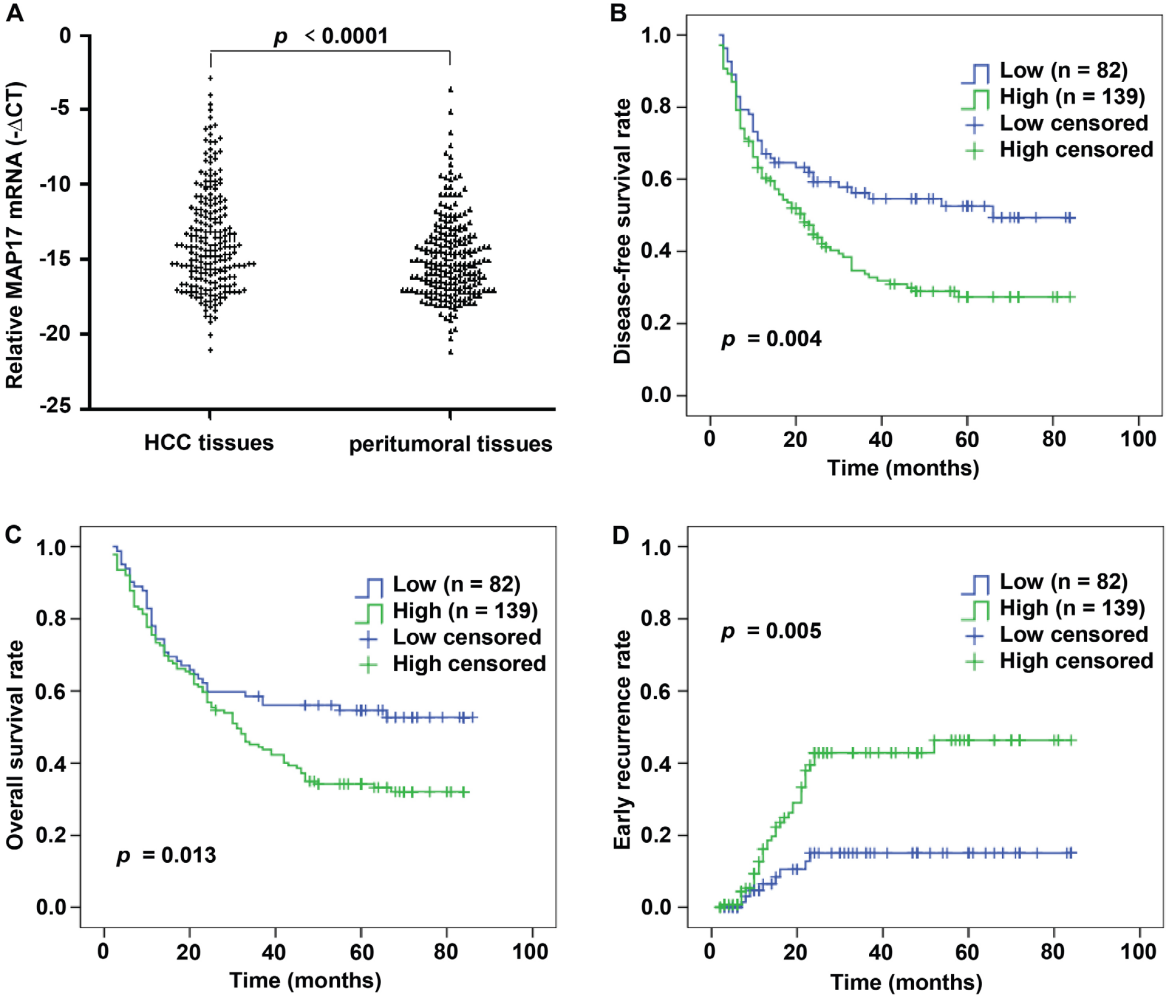
MAP17 expression is increased in HCC samples by qRT-PCR and correlated with unfavorable prognosis in HCC cohort **(A)** The mRNA levels of MAP17 were determined by qRT-PCR in 221 paired HCC and matched peritumoral tissues. For each sample, the relative mRNA level of MAP17 was normalized based on the β-actin, the scatter diagram data shown are the mean –ΔCT. **(B-D)** Kaplan-Meier analysis showed that HCC patients with high expression of MAP17 have significantly poorer DFS (B) (*p* = 0.004), OS (C) (*p* = 0.013) and high early recurrence probability (D) (*p* = 0.005) than those with low expression of MAP17.

To assess the expression of MAP17 mRNA in patients with HCC, we carried out qRT-PCR assay in 221 pairs of tumor and corresponding peritumoral samples. After normalizing to β-actin, 139 of the 221 HCC tissues (62.90%) showed a ≥ 2-fold increase of the MAP17 mRNA compared with the corresponding peritumoral tissues (*p* < 0.0001; Figure 1A). This finding confirmed that MAP17 mRNA expression is significantly increased in HCC relative to peritumoral liver tissues.

### Correlation between MAP17 expression and clinicopathologic features of HCC

To disclose the clinical significance of MAP17 in HCC, the relationships between MAP17 expression and clinicopathological parameters of HCC patients were analyzed. The results showed that high expression level of MAP17 mRNA was positively correlated with male (*p* = 0.001), distant metastasis (*p* = 0.009), early recurrence (*p* = 0.004), and high serum AFP level (> 100 ng/ml) (*p* = 0.022). However, our analyses did not show significant correlations between the expression of MAP17 and age, family history, liver cirrhosis, HBsAg, tumor size, tumor number, wine-drinking, TNM stage and serum AST level (all *p* > 0.05) (Table 2).

**Table 2:** Correlation between the clinicopathologic variables and MAP17 in HCC

Clinical character	Clinical variable	No. of patients	MAP17 mRNA		*x^2^*	*p* value
			Low n (%)	High n (%)		
Gender	Female	29	19 (65.5)	10 (34.5)	11.547	**0.001**
	Male	192	63 (32.8)	129 (67.2)		
Age (years)	≤ 55	147	57 (38.8)	90 (61.2)	0.526	0.468
	> 55	74	25 (33.8)	49 (66.2)		
Family history	No	187	74 (39.6)	113 (60.4)	3.173	0.075
	Yes	34	8 (23.5)	26 (76.5)		
HBsAg	Negative	40	13 (32.5)	27 (67.5)	0.444	0.505
	Positive	181	69 (38.1)	112 (61.9)		
Tumor size (range, cm)	≤ 5	81	29 (35.8)	52 (64.2)	0.093	0.761
	> 5	140	53 (37.9)	87 (62.1)		
Liver cirrhosis	No	20	5 (25.0)	15 (75.0)	1.381	0.240
	Yes	201	77 (38.3)	124 (61.7)		
Tumor number	Single	154	56 (36.4)	98 (63.6)	0.119	0.730
	Multiple	67	26 (38.8)	41 (61.2)		
Wine-drinking	No	110	43 (39.1)	67 (60.9)	0.370	0.543
	Yes	111	39 (35.1)	72 (64.9)		
TNM stage	I–II	94	40 (42.6)	54 (57.4)	2.081	0.149
	III–IV	127	42 (33.1)	85 (66.9)		
Distant metastasis	No	201	80 (39.8)	121 (60.2)	6.922	**0.009**
	Yes	20	2 (10.0)	18 (90.0)		
Early recurrence (≤2 year)	No	156	68 (43.6)	88 (56.4)	9.560	**0.002**
	Yes	65	14 (21.5)	51 (78.5)		
Serum AFP (ng/ml)	≤ 100	92	26 (28.3)	66 (71.7)	5.282	**0.022**
	> 100	129	56 (43.4)	73 (56.6)		
Serum AST (U/L)	≤ 40	110	39 (35.5)	71 (64.5)	0.255	0.613
	> 40	111	43 (38.7)	68 (61.3)		

HBsAg, hepatitis B surface antigen; TNM, tumor-node-metastasis; AFP, alpha-fetoprotein; AST: Aspartate aminotransferase.

We further evaluated the relationship between MAP17 and AFP through correlation analysis. The expression of MAP17 and serum AFP level did not completely overlap. The percentage was 33.03% (73/221) in MAP17 ^high^ AFP ^high^ subgroup, 25.34% (56/221) in MAP17 ^low^ AFP ^high^ subgroup, and 29.86% (66/221) in MAP17 ^high^ AFP ^low^ subgroup. These results suggested a combined detection of MAP17 and AFP in improving the sensitivity of HCC histological diagnosis to 88.24%.

### MAP17 overexpression is closely correlated with early recurrence

**Figure 2:**
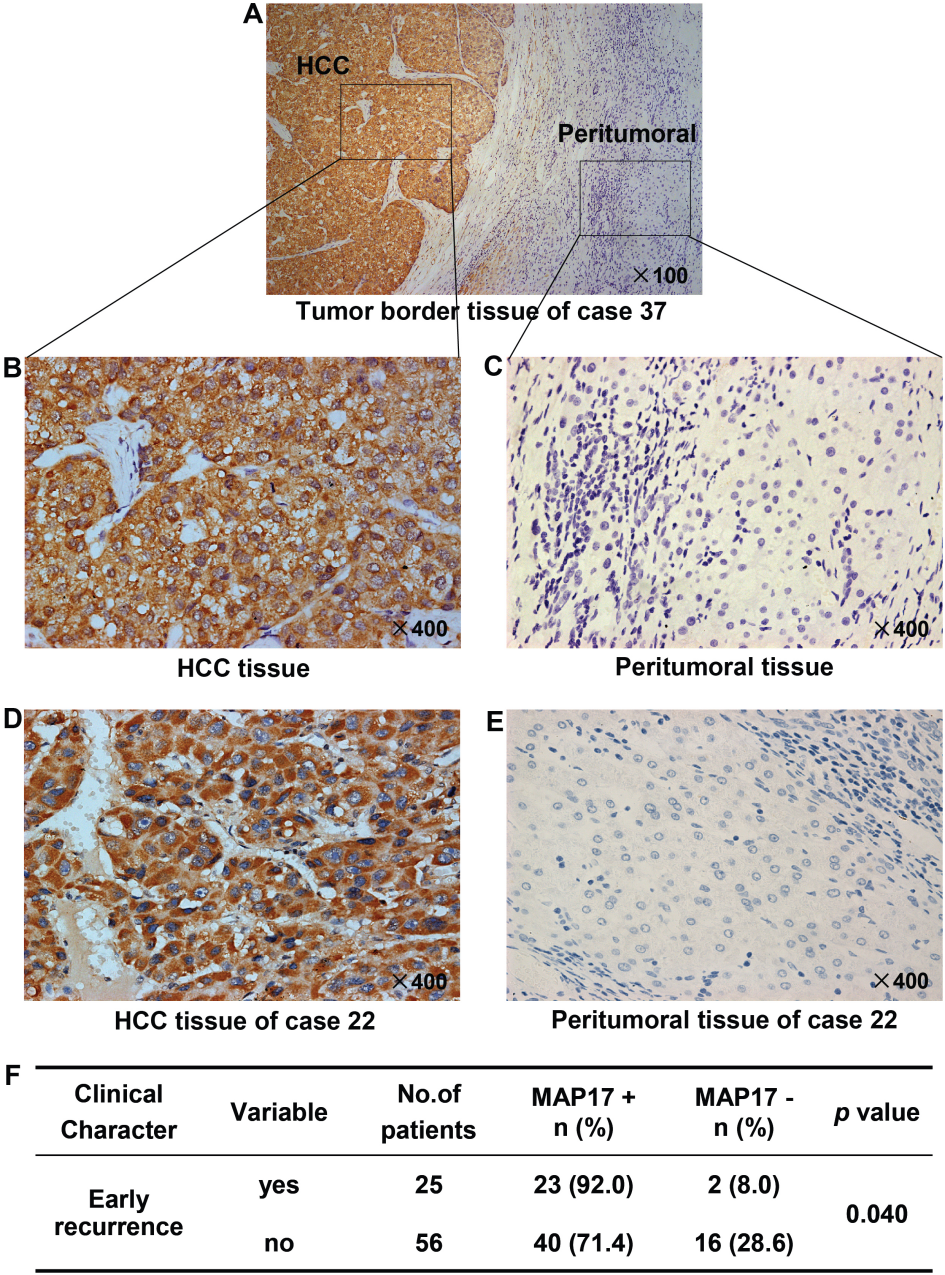
Protein expression pattern of MAP17 in HCC and paired peritumoral tissues Representative pictures are shown, the nuclei were counterstained with hematoxylin, original magnification. Tumor border tissue **(A)** of representative intratumoral high density staining **(B)** and peritumoral low density staining **(C)** of MAP17 are shown. **(D, E)** Expression of MAP17 in HCC tissue (D) and paired peritumoral tissue (E). **(F)** Statistical analysis was performed using the Fisher exact test to compare the relative levels of MAP17 negative (MAP17−) or positive (MAP17+) between HCC with early recurrence and HCC without early recurrence (*p* = 0.040).

To further confirm the expression of MAP17 at the protein level in HCC tissues, 81 pairs of paraffin-embedded HCC samples were detected by IHC, and the staining intensity was scored on a scale of 0 to 3. The proportion of positive staining of MAP17 was 80.24% (65/81) in tumor tissues, but only 22.22% (18/81) in peritumoral tissues (*p* < 0.0001). The staining patterns also showed that MAP17 was mostly anchored in the cell membrane and cytoplasm (Figure 2A-2E).

Interestingly, the MAP17 levels were significantly higher in the HCC specimens with early recurrence than in those without early recurrence (*p* = 0.040; Figure 2F), suggesting that MAP17 overexpression is provide a potential value for effectively monitoring an early recurrence of HCC after surgical radical resection.

### Elevation of MAP17 level predicted poor survival of HCC

A Kaplan-Meier survival analysis indicated that a higher mRNA expression level of MAP17 was significantly associated with a shorter DFS and OS (Figure 1B and 1C). The mean DFS in HCC patients with low MAP17 expression was 50.19 months (95% CI, 42.27-58.11), compared with 34.91 months (95% CI, 29.29-40.53) in patients with high MAP17 expression (*p* = 0.004, Figure 1B). The mean OS time was 53.59 months (95% CI, 45.84-61.34) in the low MAP17 group, whereas only 41.59 months (95% CI, 36.27-46.91) in the high MAP17 group (*p* = 0.013, Figure 1C). Importantly, the Kaplan–Meier curves also revealed that high MAP17 expression significantly associated with a higher early recurrence rate compared with low MAP17 group (*p* = 0.005, Figure 1D), suggesting a novel biomarker for screening the early recurrence in HCC patients after surgery.

### Univariate and multivariate analysis of prognostic factors

In the univariate analysis, high MAP17, large tumor size (> 5 cm), multiple tumor numbers, advanced TNM stage (III-IV), and serum AST level (> 40 U/L) were significantly associated with a shorter DFS and OS for HCC patients (all *p* < 0.05; Tables 3 and 4). These statistically significant factors were further enrolled into multivariate analysis using Cox proportional hazard model for both DFS and OS. Upon multivariate analysis, high MAP17 expression (HR: 1.710; 95% CI, 1.156-2.449; *p* = 0.012), large tumor size (> 5 cm) (HR, 2.339; 95% CI, 1.468-3.726; *p* < 0.001), and high serum AST level (> 40 U/L) (HR, 1.863; 95% CI, 1.286-2.699; *p* = 0.001) were independent prognostic factors of DFS in patients with HCC (Table 3). Meanwhile, high MAP17 (HR, 1.743; 95% CI, 1.152-2.639; *p* = 0.009), large tumor size (> 5 cm) (HR, 2.378; 95% CI, 1.490-3.797; *p* < 0.001), and elevated serum AST level (> 40 U/L) (HR, 2.014; 95% CI, 1.394-2.909; *p* < 0.001) were also identified as important independent predictors of OS for HCC patients (Table 4).

**Table 3:** Univariate and multivariate analysis of disease-free survival

Clinical character	Category	No. of patients	Univariate analysis			Multivariate analysis	
			Mean	95% CI	*p* value	HR (95% CI)	*p* value
MAP17 expression	Low	82	50.19	42.27-58.11	**0.004**	1.710 (1.156-2.449)	**0.012**
	High	139	34.91	29.29-40.53			
Gender	Female	29	51.48	37.45-65.51	0.203		
	Male	192	39.47	34.52-44.43			
Age (years)	≤ 55	147	39.08	33.34-44.83	0.393		
	> 55	74	43.60	35.40-51.81			
Family history	No	187	39.21	34.16-44.27	0.209		
	Yes	34	48.66	36.42-60.91			
HBsAg	Negative	40	40.35	29.00-51.67	0.930		
	Positive	181	40.62	35.44-45.81			
Tumor size (range, cm)	≤ 5	81	60.48	53.47-67.48	**< 0.001**	2.339 (1.468-3.726)	**< 0.001**
	> 5	140	29.05	23.66-34.39			
Liver Cirrhosis	No	20	33.18	18.51-47.85	0.308		
	Yes	201	41.35	36.40-46.31			
Tumor number	Single	154	46.84	41.20-52.48	**< 0.001**	1.332 (0.917-1.935)	0.133
	Multiple	67	26.00	18.57-33.42			
Wine-drinking	No	110	45.28	38.26-52.29	0.079		
	Yes	111	36.41	30.21-42.62			
TNM stage	I–II	94	56.43	49.54-63.33	**< 0.001**	1.324 (0.906-1.931)	0.145
	III–IV	127	28.87	23.29-34.46			
Distant metastasis	No	201	41.25	36.30-46.19	0.450		
	Yes	20	30.56	18.37-42.76			
Serum AFP (ng/ml)	≤ 100	92	44.53	37.32-51.75	0.127		
	> 100	129	37.83	31.66-44.01			
Serum AST (U/L)	≤ 40	110	53.21	46.46-59.96	**< 0.001**	1.863 (1.286-2.699)	**0.001**
	> 40	111	28.79	23.07-34.52			

HR, hazard ratio; CI, confidence interval; HBsAg, hepatitis B surface antigen; TNM, tumor-node-metastasis; AFP, alpha-fetoprotein; AST: aspartate aminotransferase.

**Table 4:** Univariate and multivariate analysis of overall survival

Clinical character	Category	No. of patients	Univariate analysis			Multivariate analysis	
			Mean	95% CI	*p* value	HR (95% CI)	*p* value
MAP17 expression	Low	82	53.59	45.84-61.34	**0.013**	1.743 (1.152-2.639)	**0.009**
	High	139	41.59	36.27-46.91			
Gender	Female	29	55.96	43.49-68.42	0.079		
	Male	192	44.84	40.02-49.65			
Age (years)	≤ 55	147	44.86	39.21-51.06	0.318		
	> 55	74	49.08	41.55-56.72			
Family history	No	187	44.87	39.97-49.76	0.134		
	Yes	34	54.09	42.74-65.48			
HBsAg	Negative	40	46.63	35.86-57.48	0.897		
	Positive	181	45.68	40.85-50.56			
Tumor size (range, cm)	≤ 5	81	65.52	59.66-71.35	**< 0.001**	2.378 (1.490-3.797)	**< 0.001**
	> 5	140	34.86	29.37-40.12			
Liver Cirrhosis	No	20	38.45	24.36-52.55	0.316		
	Yes	201	46.41	41.76-51.08			
Tumor number	Single	154	52.76	47.45-58.08	**< 0.001**	1.335 (0.918-1.941)	0.130
	Multiple	67	31.54	24.18-39.11			
Wine-drinking	No	110	49.82	43.33-56.32	0.053		
	Yes	111	43.22	36.19-48.12			
TNM stage	I–II	94	62.43	56.26-68.61	**< 0.001**	1.250 (0.896-1.863)	0.231
	III–IV	127	34.06	28.58-39.53			
Distant metastasis	No	201	45.69	41.09-50.38	0.868		
	Yes	20	45.73	32.19-59.32			
Serum AFP (ng/ml)	≤ 100	92	50.59	44.10-57.13	0.094		
	> 100	129	42.92	36.83-48.96			
Serum AST (U/L)	≤ 40	110	58.62	52.41-64.86	**< 0.001**	2.014 (1.394-2.909)	**< 0.001**
	> 40	111	34.12	28.51-39.86			

HR, hazard ratio; CI, confidence interval; HBsAg, hepatitis B surface antigen; TNM, tumor-node-metastasis; AFP, alpha-fetoprotein; AST: aspartate aminotransferase.

### MAP17 promoted the malignant progression of HCC cells *in vitro*

**Figure 3:**
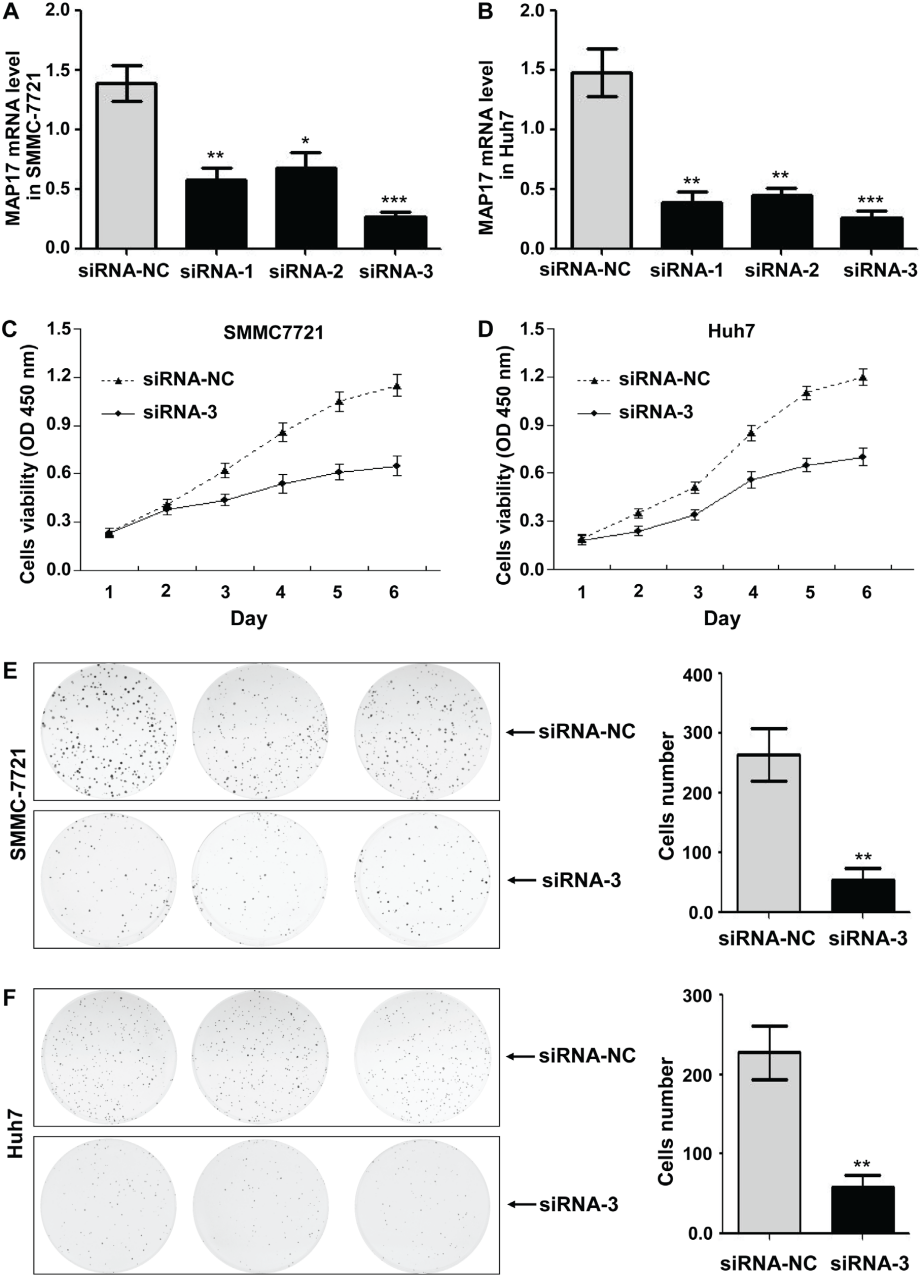
The effect of MAP17 on the growth and proliferation of HCC cells *in vitro* **(A, B)** The silent effect of siRNA was assessed by qRT-PCR in SMMC7721 (A) and Huh7 (B). **(C, D)** The cell growth curves after downregulation of MAP17 were determined by the CCK-8 assay in SMMC7721 (C) and Huh7 (D). SiRNA-NC was used as control. **(E, F)** Clone formation assay was performed in SMMC7721 (E) and Huh7 cells (F), with siRNA-NC serving as a control. Histograms represent the mean numbers of colonies obtaining from triplicate tests (mean±SD). (^*^, P < 0.05; ^**^, P < 0.01; ^***^, P < 0.001).

**Figure 4:**
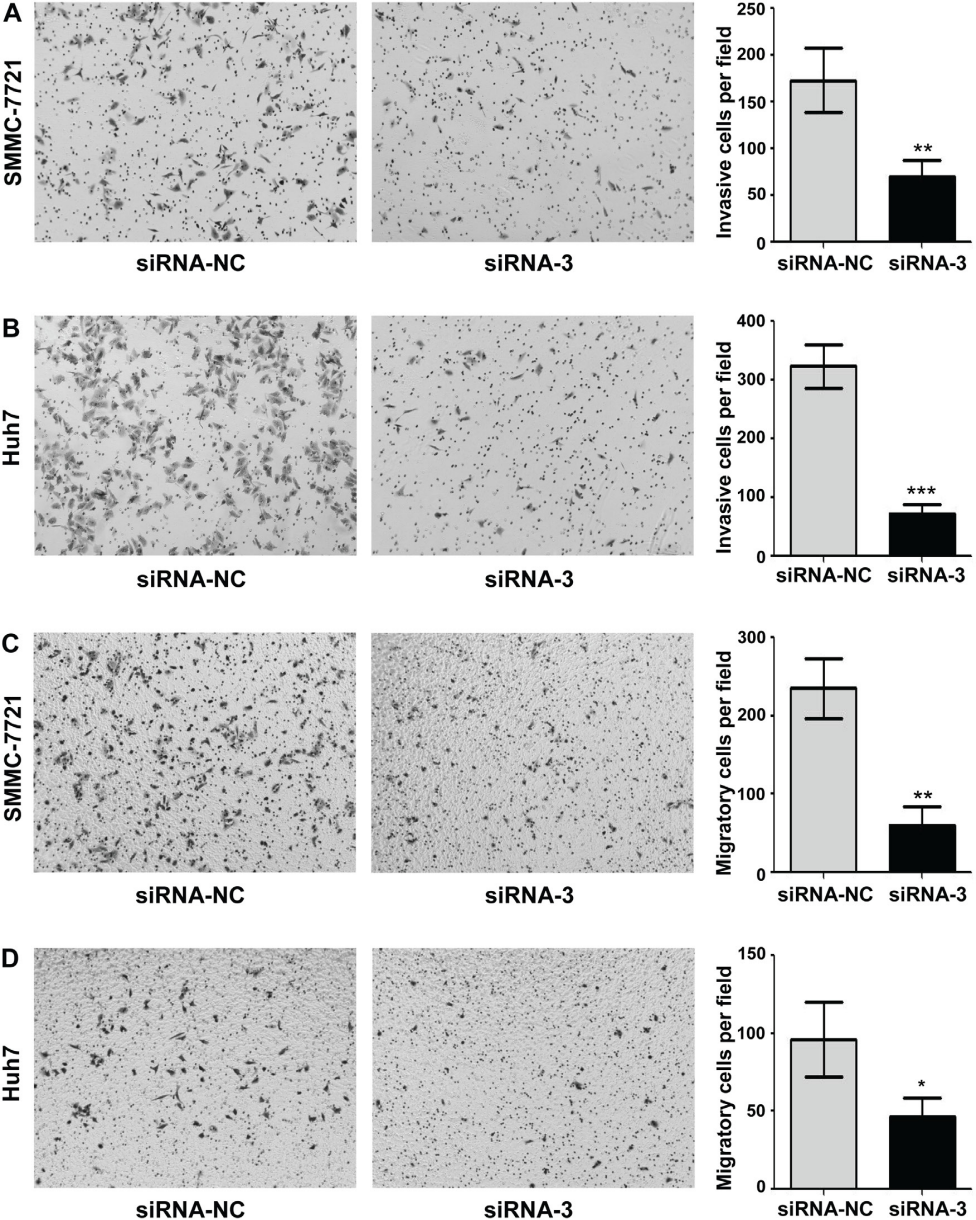
Knockdown of MAP17 inhibited the invasion and migration of HCC cells *in vitro* **(A, B)** Silencing of MAP17 significantly decreased the invasion ability of SMMC7721 (A) and Huh7 (B) cells compared with control group. **(C, D)** Transwell migration assay illustrated that knockdown of MAP17 significantly reduced the migration of SMMC7721 (C) and Huh7 (D) compared with control group. The histograms represent the mean values of invasive and migrated cells per field (from at least 5 fields, mean ± SD) (^*^, P < 0.05; ^**^, P < 0.01; ^***^, P < 0.001).

To further investigate the biological function of MAP17 on HCC cells, we used the MAP17-specific siRNA to knock down endogenous MAP17 expression in HCC cells and compared the changes in cellular biological behaviors. The effect of silencing MAP17 in SMMC7721 and Huh7 cells were evaluated by qRT-PCR, with siRNA-NC served as a reference (Figure 3A and 3B). As shown in Figure 3C and 3D, knockdown of MAP17 could significantly inhibit the growth of SMMC7721 and Huh7 cells. Similarly, downregulation of MAP17 also restrained the proliferation of both SMMC7721 and Huh7 cells through the clone formation assay (Figure 3E and 3F). In addition, cell invasion assay illustrated that silencing of MAP17 significantly decreased the invasive ability of SMMC7721 and Huh7 cells (*p* < 0.01 and *p* < 0.001, respectively, Figure 4A and 4B). Transfection of siRNA-MAP17 in SMMC-7721 and Huh7 cells also obviously reduced cell migration compared with the control group (*p* < 0.01 and *p* < 0.05, respectively, Figure 4C and 4D). These results reveal that MAP17 may play an important role in promoting the malignant progression of HCC cells.

### MAP17 regulated the Akt/mTOR signaling pathway in HCC cells

**Figure 5:**
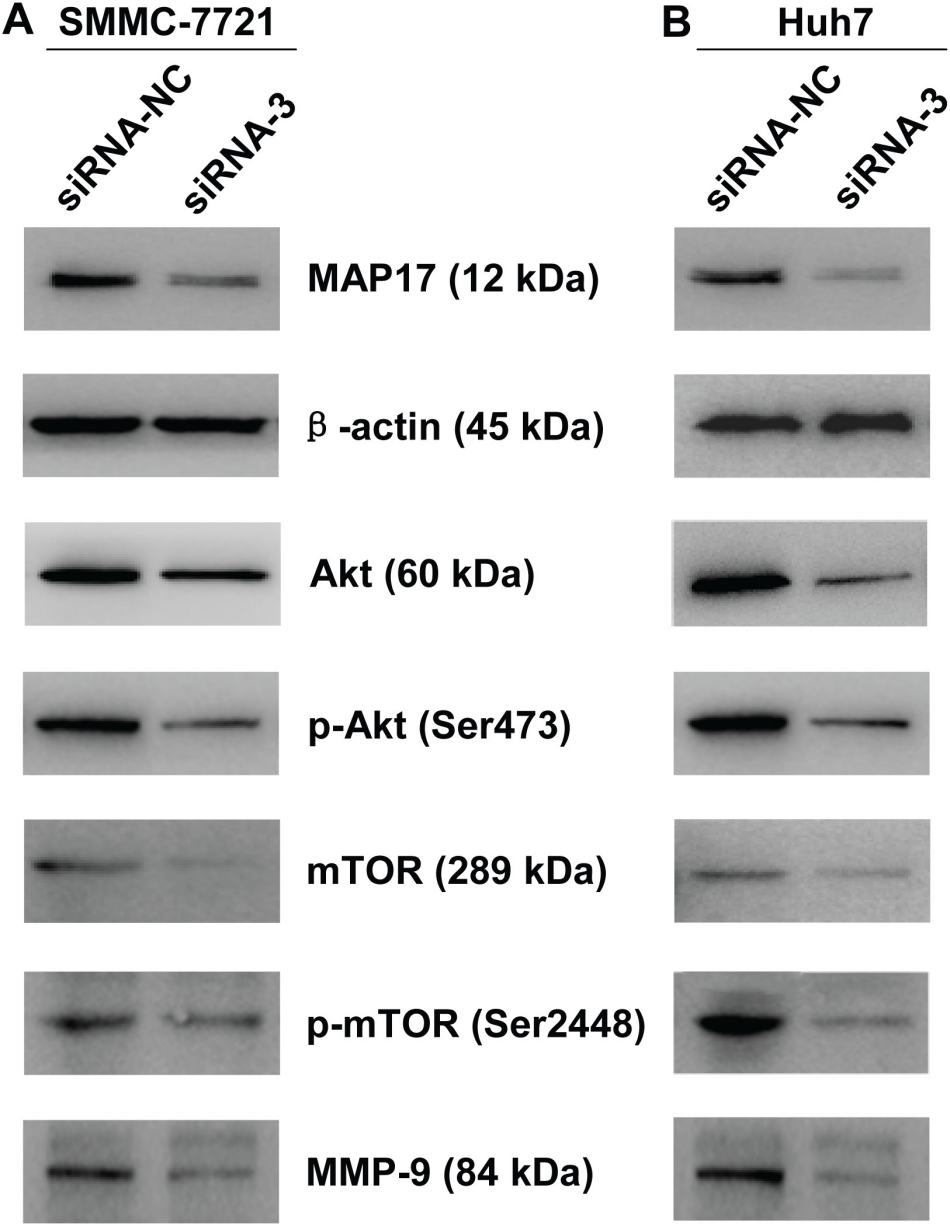
Downregulation of MAP17 inhibited Akt/mTOR signaling pathway in HCC cells Downregulation of MAP17 inhibited the phosphorylation of Akt (Ser473) and mTOR (Ser2448) and decreased expression levels of Akt, mTOR and MMP-9 in SMMC7721 (A) and Huh7 (B). β-actin was used as a reference.

To explore the potential molecular mechanism of MAP17 in promoting HCC cells progression, western blot analysis was performed to detect the signal pathways that may play important roles in the proliferation, invasion and migration of HCC cells. The results showed that knockdown of MAP17 significantly decreased the expression levels of Akt and p-Akt (Ser473) in both SMMC7721 and Huh7 cells (Figure 5A and 5B). Meantime, the expression levels of Akt downstream related proteins, including mTOR, p-mTOR (Ser2448) and MMP-9, were also reduced after silencing MAP17 (Figure 5A and 5B). Consequently, these results indicated that MAP17 may promote HCC cells malignant progression by the Akt/mTOR signaling pathway.

## DISCUSSION

In this study, we found that MAP17 expression was significantly up-regulated in HCC tissues at both mRNA and protein level. Previous researches have reported the enhanced tumorigenic properties induced by MAP17 are functionally related to the ROS production in tumor cells [15]. Cancer cells can develop an increasing constitutive oxidative stress to promote tumor growth and protect them from oncogene-induced pre-apoptotic, death and senescence events, thus rapidly activated signaling cascades can promote tumorigenic processes [16, 17]. As a signal transduction messenger, ROS participate in and regulate sustained proliferation and transformation of carcinoma cells [17]. On one hand, ROS can immediately modify signaling proteins by various modifications, including nitrosylation, carbonylation, disulfide bond formation and glutathionylation in signaling cascades [18]. On the other hand, ROS can reprogram the expression of enzymes and other proteins in the proximal target cells [19]. The Na-dependent glucose transporter 1 (SGLT1) membrane transport mediated MAP17-induced ascending trend of endogenous ROS, resulted in the activation of growth-related, genetic program in malignant cells [20]. Therefore, combined with aforementioned studies, it may be speculated that HCC cells may take advantage of MAP17 overexpression to elevate the level of endogenous ROS, leading to initiation and/or progression of the HCC.

Recurrence is an important obstacle to enhance the outcome of patients with HCC, especially early recurrence [21]. Recently, researchers tend to deem that metastasis and recurrence of HCC are independent of the time of tumorigenesis or tumor size, but depend on the potential of invasion and metastasis of HCC cells, which has been obtained in the early stage of primary cancer (small HCC stage). Nevertheless, not all of the cancer cells share the same properties within a tumor, and this kind of heterogeneous hierarchical structure is an important feature of tumor [22]. Thus, only part of the HCC cells own the ability to invasion and metastasis. In the study, it was observed that high MAP17 expression was positively correlated with distant metastasis and early recurrence. Meanwhile, MAP17 protein was overexpressed in HCC patients with early recurrence, but not in those without early recurrence. Therefore, the subset of MAP17-positive cells may be a subgroup with strong metastasis and recurrence potentials in HCC, which may indicate a new idea for therapeutic target. It is noteworthy that patients with high MAP17 expression significantly associated with a higher early recurrence rate. Hence, MAP17 can help identify patients who are prone to early recurrence. In addition, MAP17 expression was strongly correlated with AFP. When combined both of MAP17 and AFP as biomarkers, the positive diagnosis rate of HCC was significantly improved. Accordingly, MAP17 may serve as a novel biomarker for the diagnosis and early recurrence of HCC.

High MAP17 expression was significantly associated with a shorter DFS and OS. Upon multivariate analysis, MAP17 was also an important independent predictor of OS and DFS for patients with HCC. Except for MAP17, FAM83D [23] was conformed as independent predictors of OS and DFS for HCC patients in our previous studies. However, considering that the clinical diagnosis and predictive value of single gene has certain limitations, it is great value to construct multi-molecular model. Recently, Ji et al reported that tumor gene signatures can help stratify patients in regard to prognosis and response to the therapy [24]. Prognosis estimates for certain malignancies have been successfully improved by molecular classification [25, 26]. Yet molecular model for accurately predicting prognosis in HCC patients has long been discouraged by the complexity of molecular basis. Therefore, the MAP17 and FAM83D are available to help construct a best predictive model for predicting postoperative metastases/recurrence, outcome, or even response to the therapy for HCC patients. In addition, consistent with previous studies, large tumor size was an independent predictor of OS and DFS for HCC patients [27, 28]. HCC patients with a larger tumor size were more vulnerable to suffer vascular invasion and early recurrence disease, thus showed a significantly higher risk of death [29, 30].

The results of cell functional studies indicated that MAP17 promoted the proliferation, invasion and migration of HCC cells. But the underlying mechanism is still unclear. Accumulating evidences demonstrate that Akt/mTOR signaling pathway plays a crucial role in cancer progression. In this study, western blot results showed that knockdown of MAP17 can downregulate the expression levels of related proteins of Akt/mTOR signaling pathway, such as Akt, p-Akt (Ser473), mTOR, p-mTOR (Ser2448) and MMP-9. These results suggest that MAP17 promotes the malignant progression of HCC cells by Akt/mTOR signaling pathway.

In conclusion, MAP17 was up-regulated in HCC tissues and was an independent prognostic factor for patients with HCC. The elevation of MAP17 expression level was significantly associated with a higher early recurrence rate of HCC after surgery. Up-regulation of MAP17 promoted HCC cells proliferation, invasion, and migration through Akt/mTOR signaling pathway. Thus, MAP17 may serve as a promising biomarker for the diagnosis of HCC and predicting the risk of early recurrence and survival after surgery.

## MATERIALS AND METHODS

### Ethics statement

Ethical approval for our study was obtained from the institutional ethics committee of the Affiliated Hospital of Guilin Medical University. All samples used for research purposes have prior written informed consents from the participating patients, and the procedures strictly followed the ethics guidelines of the 1975 Declaration of Helsinki.

### Patient and tissue samples

221 paired primary HCC tumors and adjacent peritumoral tissues along with the clinicopathological information were collected from HCC patients after surgical resection at the Affiliated Hospital of Guilin Medical University, Guilin, Guangxi, China between May 2001 and June 2008. All fresh tumor tissues were immediately snap frozen in liquid nitrogen and kept at –80°C until using it. The archival formalin-fixed paraffin-embedded (FFPE) HCC tissues were cut into 3μm thick slices and mounted on microscope slides for further investigation. All HCC specimens were accurately diagnosed based on histopathological examination. Tumor stage was determined according to the 7th edition tumor-node-metastasis (TNM) classification of the International Union Against Cancer [31]. Demographic variables and clinical features of these patients, including age, gender, alcohol abuse, liver cirrhosis, hepatitis B surface antigen (HBsAg), tumor size, alpha-fetoprotein (AFP), white blood cell, platelets, albumin, globuline, total bilirubin, direct bilirubin, alanine transaminase, aspartate aminotransferase (AST), alkaline phosphatase, γ-glutamyltransferase, family history, tumor number, TNM stage, distant metastasis and early recurrence were retrieved and presented in Tables 1 and 2.

The definition of an early recurrence is that tumor recurs within 2 years after surgical radical HCC resection. To monitor recurrence, all HCC patients were assessed by routine clinical examination at regular intervals. Abdominal ultrasonography (US), chest radiography and serum AFP testing were conducted every 2 months in the first 2 years, and every 3 to 6 months thereafter. Dynamic computed tomography or magnetic resonance imaging was performed when the serum AFP testing showed an increasing trend or the US indicated the appearance of possible malignant lesions. The disease-free survival (DFS) was defined as a time span from the date of surgery to the date of recurrence, metastasis, death or the last follow-up. Overall survival (OS) was calculated from the date of surgery to the date of death or last contact. The cut-off value between early and late recurrence was set as 2 years.

### RNA extraction and qRT-PCR assay

Total RNA was extracted from frozen clinical samples using the TRIzol reagent (Invitrogen, Carlsbad, CA, USA), and was reverse-transcribed using a Prime Script RT Reagent Kit (TaKaRa, Otsu, Japan) following the manufacturer's instructions. Real-time PCR was performed to detect the expression levels of MAP17 and the corresponding endogenous control β-actin using the ABI Prism 7500 Sequence Detector System (Applied Biosystems, Foster City, CA, USA) with a 20 μl reaction volume containing 1 μl of cDNA template, 10 μl of SYBR Green PCR Master Mix (Applied Biosystems, Foster City, CA, USA), 0.2 μl of forward primer and reverse primer respectively and 8.6 μl of nuclease-free water. Primer sequences for MAP17 and β-actin were as follows: MAP17, forward: 5′-CTGCACACATGATCCTGACCG-3′ and reverse: 5′-CTCACTGGACCTGAAACTGGC-3′; β-actin, forward: 5′- GACAGGATGCAGAAGGAGATTACT-3′ and reverse: 5′- TGATCCACATCTGCTGGAAGGT-3′. The PCR cycling began with template denaturation at 95°C for 10 mins, and then 40 cycles of 95°C for 30 secs, 55°C for 30 secs and 72°C for 30 secs, and finally an extension at 72°C. Relative quantification of MAP17 mRNA was performed as previously described [23], and data were normalized to β-actin (an internal control). All samples were performed in triplicate to assure the results.

### IHC analysis

In order to clear away the paraffin around, all FFPE HCC specimens were treated with xylene, then rehydrated in a series of decreasing concentrations of alcohol. Next, these samples were boiled in citrate antigenic retrieval buffer (pH = 6.0) for 3 minutes using a pressure cooker and cooled to room temperature, and then endogenous peroxidase activity was quenched by 3% H_2_O_2_ for 10 mins at room temperature. Then, the sections were treated with 10% goat serum at room temperature for 30 mins in order to prevent non-specific binding. After that, the sections were incubated with rabbit monoclonal MAP17 antibody (ab156014, Abcam Company) diluted in a working solution (1:200) at 4°C for a night, washed in phosphate buffered saline (PBS), incubated with biotinylated secondary antibody at room temperature for 30 mins, and exposed to streptavidin-horseradish peroxidase complex at room temperature for 20 mins. The sections were developed with 3, 3- diaminobenzidine tetrahydrochloride and counterstained with hematoxylin. As a negative control, the primary antibody was replaced by an irrelevant rabbit serum under the same experimental conditions. The expression levels of MAP17 protein in samples were independently evaluated by semi-quantitative IHC method by two pathologists who were blinded to the clinical and follow-up data of HCC patients. The proportion of tumor cells was scored as follows: grades 0 to 3 (0, no positive cell; 1, < 25% positive cells; 2, 25%-50% positive cells; 3, > 50% positive cells).

### Cell culture

SMMC7721 and Huh7 cells were obtained from Institute of chemistry and cell biology (Shanghai, China) and were cultured in Dulbecco's modified Eagle's medium (DMEM; Gibco, Grand Island, NY, USA) containing with 10% fetal bovine serum (FBS), at 37°C and 5% CO_2_ incubator.

### Transient transfection

Three siRNAs against MAP17 were designed and synthesized by GenePharma Con., Ltd (Shanghai). The sequences of three pairs of siRNAs were as follows: siRNA-1: 5′-GCAGAUGGAGUCCUGGUGGTT-3′ and 5′-CCACCAGGACUCCAUCUGCTT-3′; siRNA-2: 5′-CCAGGCACAUGGGAUGGAUTT-3′, and 5′-AUCCAUCCCAUGUGCCUGGTT-3′; siRNA-3: 5′-CCAGUUUCAGGUCCAGUGATT-3′, and 5′-UCACUGGACCUGAAACUGGTT-3′. The siRNA-NC (5′-GAGUUAAAGUCAAAGUGACTT-3′, 5′-GUCACUUUGACUUUAACUCTT-3′) was used as negative control. In serum-free medium conditions, SMMC7721 and Huh7 cells were transfected with siRNA by using Lipofectamine™ 3000 (Invitrogen) according to the manufacturer's instructions for 8 hours, then changed with DMED containing 10% FBS and continuously cultured for 36 hours.

### Clone formation and viability assay

Transfected cells were inoculated in 6-well plates and cultured with 2 ml DMEM for two weeks. Then the cells were fixed with 4% paraformaldehyde for 1 hour and dyed with 0.1% crystal violet over night. After washing with PBS for three times, cell colonies were photographed and counted.

For viability assay, the cells were seeded in 96 wells plate and each well contained 5000 cells in 100μl DMEM. The cell viability was measured using the cell counting kit-8 (CCK-8) according to the manufacturer's instructions, and the experimental procedures were performed as previously described [23].

### Cell invasion and migration assay

Cell invasion was performed using Matrigel Invasion Chamber (CORNING, USA) and cell migration was performed using 8 μm pore size Transwell chambers (CORNING, USA). In brief, 4×10^4^ cells were cultured in the upper chamber with serum-free medium, and the lower chamber filled with DEME contained 20% FBS. After 24 hours of incubation, the adherent cells in the inner surface of chamber were wiped with cotton swab. The invasive and migrated cells were fixed with 4% paraformaldehyde for 30 minutes, stained with 0.1% crystal violet overnight, and then photographed with inverted microscope.

### Western blotting

After transfection, the total cell protein was extracted by using lysis buffer (Beyotim Biotechnology) containing with protease inhibitor. Then protein samples were separated by 8-15% SDS-PAGE at 90 Volts and transferred to polyvinylidene difluoride (PVDF) membranes (BioRad) at 250 mA electric current. The membranes were blocked with 5% skim milk for 1 hour and incubated with the primary antibody overnight at 4°C. The primary antibody were used as follows: MAP17 antibody (ab156014, Rabbit monoclonal antibody, 12 kDa, dilution 1:1000, Abcam Company), Akt antibody (#4685, rabbit monoclonal antibody, 60 KDa, dilution 1:1000, Cell Signaling Technology), phospho-Akt (Ser473) antibody (#12694, rabbit monoclonal antibody, 60 KDa, dilution 1:1000, Cell Signaling Technology), mTOR antibody (#2972, rabbit polyclonal antibody, 289 KDa, dilution 1:1000, Cell Signaling Technology), phospho-mTOR (Ser2448) antibody (#2971, rabbit polyclonal antibody, 289 KDa, dilution 1:1000, Cell Signaling Technology), MMP-9 antibody (#13667, rabbit monoclonal antibody, 84 KDa, dilution 1:1000, Cell Signaling Technology), β-actin (#12620, rabbit monoclonal antibody, 45 KDa, dilution 1:1000, Cell Signaling Technology). After washing three times with TBST (Tris buffered saline plus 0.1% Tween 20), the membranes were incubated with secondary antibody, and then exposed on X-ray films using BeyoECL Plus (Beyotim Biotechnology).

### Statistical analysis

All statistical analyses were performed using the SPSS 18.0 (SPSS Inc, Chicago, IL). The difference of MAP17 mRNA expression between HCC tissue and the peritumoral tissue was compared using the nonparametric Tests. The association between MAP17 expression levels and other clinicopathological features was analyzed using the Pearson χ^2^ test or the Fisher exact test. Kaplan-Meier analysis and the log-rank test were used to estimate the DFS and OS. The prognostic significance of MAP17 and other clinicopathologic features were identified using the Cox proportional hazards regression model. A difference was statistically significant when *p* value was less than 0.05.

## Abbreviations

HCC: hepatocellular carcinoma
ROS: reactive oxygen species
qRT-PCR: quantitative real-time PCR
IHC: immunohistochemistry
FFPE: formalin-fixed paraffin-embedded
TNM: tumor-node-metastasis
HBsAg: hepatitis B surface antigen
AFP: alpha-fetoprotein
AST: aspartate aminotransferase
US: abdominal ultrasonography
DFS: disease-free survival
OS: overall survival
DMEM: Dulbecco's modified Eagle's medium
FBS: fetal bovine serum
CCK-8: cell counting kit-8
PVDF: polyvinylidene difluoride
